# Can *in vitro* embryo production be estimated from semen variables in Senepol breed by using artificial intelligence?

**DOI:** 10.3389/fvets.2023.1254940

**Published:** 2023-09-21

**Authors:** Suzane Peres Campanholi, Sebastião Garcia Neto, Gabriel Martins Pinheiro, Marcelo Fábio Gouveia Nogueira, José Celso Rocha, João Diego de Agostini Losano, Adriano Felipe Perez Siqueira, Marcílio Nichi, Mayra Elena Ortiz D'Avila Assumpção, Andréa Cristina Basso, Fabio Morato Monteiro, Lindsay Unno Gimenes

**Affiliations:** ^1^Departamento de Patologia, Reprodução e Saúde Única, Faculdade de Ciências Agrárias e Veterinárias (FCAV), Universidade Estadual Paulista, Jaboticabal, Brazil; ^2^Senepol 3G, Barretos, Brazil; ^3^Departamento de Ciências Biológicas, Faculdade de Ciências e Letras (FCLA), Universidade Estadual Paulista (UNESP), Assis, Brazil; ^4^Departamento de Reprodução Animal, Faculdade de Medicina Veterinária e Zootecnia (FMVZ), Universidade de São Paulo (USP), São Paulo, Brazil; ^5^In Vitro Brasil SA, Mogi Mirim, Brazil; ^6^Centro Avançado de Pesquisa de Bovinos de Corte, Agência Paulista de Tecnologia dos Agronegócios/Instituto de Zootecnia (APTA/IZ), Sertãozinho, Brazil

**Keywords:** bovine, sperm kinetics, artificial intelligence, fertility, IVEP

## Abstract

Thoroughly analyzing the sperm and exploring the information obtained using artificial intelligence (AI) could be the key to improving fertility estimation. Artificial neural networks have already been applied to calculate zootechnical indices in animals and predict fertility in humans. This method of estimating the results of reproductive biotechnologies, such as *in vitro* embryo production (IVEP) in cattle, could be valuable for livestock production. This study was developed to model IVEP estimates in Senepol animals based on various sperm attributes, through retrospective data from 290 IVEP routines performed using 38 commercial doses of semen from Senepol bulls. All sperm samples that had undergone the same procedure during sperm selection for *in vitro* fertilization were evaluated using a computer-assisted sperm analysis (CASA) system to define sperm subpopulations. Sperm morphology was also analyzed in a wet preparation, and the integrity of the plasma and acrosomal membranes, mitochondrial potential, oxidative status, and chromatin resistance were evaluated using flow cytometry. A previous study identified three sperm subpopulations in such samples and the information used in tandem with other sperm quality variables to perform an AI analysis. AI analysis generated models that estimated IVEP based on the season, donor, percentage of viable oocytes, and 18 other sperm predictor variables. The accuracy of the results obtained for the three best AI models for predicting the IVEP was 90.7, 75.3, and 79.6%, respectively. Therefore, applying this AI technique would enable the estimation of high or low embryo production for individual bulls based on the sperm analysis information.

## 1. Introduction

In traditional breeding, a soundness evaluation usually identifies bulls with substantial fertility deficits. However, despite many animals presenting apparently normal ejaculate during routine evaluations, the fertility rates are reduced (1, 2). The rejection and approval of semen doses after routine evaluation performed in most semen processing centers differs from the results of the evaluation performed using computer-assisted sperm analysis (CASA) and flow cytometry, which directly reflect in the reproductive indices of the cattle (3).

Laboratory tests alone cannot accurately predict the fertilizing ability of semen samples. The evaluation of a combination of sperm characteristics *in vitro* can better predict semen quality than can the evaluation of a single parameter alone (4, 5). In cattle, various sperm characteristics are related to *in vitro* fertility. Previous studies that evaluated sperm morphology (6–8), sperm subpopulations (9, 10), plasma and acrosomal membrane integrity (11–13), mitochondrial membrane potential (12), oxidative status (14, 15) and DNA (16, 17) suggest that these assessments can define the efficiency of *in vitro* embryo production (IVEP). This biotechnology was responsible for generating 93.8% of bovine embryos in Brazil in 2019 (18).

A limitation of studies that evaluated different sperm characteristics with the aim to establish a relationship between the characteristics and fertility is that most of them underestimated the amount of information obtained in semen evaluations when performing classical univariate statistical analysis on a variable-by-variable basis. Assessments that encompass several characteristics at the same time better represent the physiological reality of animals, as many attributes are interdependent; therefore, they should not be considered in isolation. The power to predict fertility could be increased when a multivariate assessment is performed (19).

An interesting option for studying non-linear relationships compared with that of traditional statistical methods is the application of artificial neural network (ANN) analysis. ANN is a type of artificial intelligence (AI), which learn through the training modality similar to how the human brain learns, assimilates, and remembers information in anticipation of future events (20). In animals, this analysis has already been applied to estimate or predict zootechnical indices, such as growth curves, birth weight, milk production, and egg production (21–27). Hence, the application of biotechnological methodology could provide major improvements for livestock in our country. Furthermore, using this type of AI in animal andrology opens the opportunity for new applications of this analysis in other areas of veterinary medicine and scientific research.

Therefore, the aim of this study was to verify whether data from sperm subpopulations and other sperm parameters from Senepol bulls would be predictive for *in vitro* embryo production when using trained software based on artificial intelligence. We hypothesize that the trained program would be effective (to retrospectively predict high or low embryo production) above 70% accuracy.

## 2. Material and methods

### 2.1. Ethics committee

This study was approved by the Animal Ethics Commission of FCAV/UNESP, Jaboticabal, on August 18, 2016 (Protocol No. 12,807/16).

This manuscript is a continuum of the study “Estimate of *in vitro* embryo production based on sperm subpopulations in Senepol bulls” published by our research group in the journal *Theriogenology*. More specific details of the methodology can be obtained from the paper by Campanholi et al. (9).

### 2.2. Collection of embryo yield data

Retrospective data from 290 IVEP routines, which generated 2,361 Senepol embryos, were obtained from the records of Senepol 3G. All routines were performed in the same commercial laboratory (*In vitro* Brazil, Mogi Mirim, SP, Brazil) by following the protocol reported by Morotti et al. (28).

### 2.3. Sample selection, conditioning and thawing

Semen samples were chosen based on the presence of complete data (donor and bull identification, batch, seminal sample identification, number of viable oocytes, and embryos) from at least two IVEP routines. After assessing the availability of doses at Senepol 3G, 38 cryopreserved semen samples from 28 Senepol bulls were selected. Each sample was related to a specific bull batch used in IVEP routines. The mean (± standard error of the mean) for the IVEP routines of the semen samples used was 26.55 ± 0.89 and that for viable oocytes was 33.99 ± 1.21.

The straws were stored in a cryogenic cylinder with liquid nitrogen (−196°C). At the time of analysis, they were thawed in a water bath at 37°C for 45 s, according to the procedures of the commercial laboratory IVEP.

### 2.4. Semen washing and analysis

The detailed methodology of this study was previously described by Campanholi et al. (9). Briefly, all samples were thawed and underwent specific Senepol bull semen washing prior to *in vitro* fertilization (IVF), according to the commercial laboratory of IVEP. The washing process consisted of two washes with sperm-TALP (Tyrode albumin lactate pyruvate).

Immediately after thawing, an aliquot of semen was separated for sperm morphology analysis using a wet preparation technique. All washed samples were subjected to individual sperm kinetics analysis using CASA, according to the methodology previously described by Ferraz et al. (10).

Analysis of plasma and acrosomal membrane integrity, mitochondrial membrane potential, oxidative status, and chromatin resistance was performed using flow cytometry after washing. Plasma and acrosomal membrane integrity were evaluated simultaneously using propidium iodide (PI) and fluorescein isothiocyanate-conjugated *Pisum sativum* [FITC-PSA; (12)]. Mitochondrial membrane potential was evaluated using tetraethylbenzimidazole carbocyanine iodide [JC-1; (12)]. Oxidative status was analyzed according to the protocol described by Castro et al. (14). Chromatin resistance analysis was performed based on the sperm chromatin structure assay [SCSA; (12)].

### 2.5. Statistical analysis

As described previously by Campanholi et al. (9), multivariate statistical analyses were performed to characterize the sperm subpopulations: subpopulation 1 (SBP1) is characterized by fast and progressive sperm motility; subpopulation 2 (SBP2) is characterized by fast and non-progressive sperm motility, which can configure a hyperactivation movement; and subpopulation 3 (SBP3) is characterized by slow and non-progressive sperm motility. To study the relationship between the three sperm subpopulations and other semen variables associated with IVEP, the response variable embryo production was categorized based on the analysis of the IVEP dataset regarding the Senepol breed (29–31). In the present study, only two categories were used: high embryo yield (rates between 30–60%) and low embryo yield (rates between 0–19.99%).

In this study, a supervised learning technique was used to train the AI algorithm. This allows the classification algorithm to be evaluated using a confusion matrix. A confusion matrix enables a comparison between the true value of an instance and its output obtained by the classification model (32). This method allows the quantification of true-positive, true-negative, false-positive, and false-negative classifications. From these metrics, we analyze a graph plot of the true positive (y-axis) and false negative (x-axis) classes called the receiver operating characteristic [ROC; (33)].

To facilitate a comparison of the efficiencies of the AI algorithms, we calculated the area under the ROC curve (AUC). AUC is a scalar measure between 0 and 1, which represents the probability that the classifier will rank a randomly chosen positive instance higher than a randomly chosen negative instance (34). The closer the AUC value is to 1, the better the generalization and classification capability of the AI algorithm.

### 2.6. Artificial intelligence

For the application of the AI technique, we used ANNs of the multilayer perceptron type (35, 36), whose architecture is composed of an initial layer, intermediate layers, and output layer. In this study, the initial layer was composed of 18 input variables, namely SBP1, SBP2, SBP3, major defects (DMA), minor defects (DMI), total defects (TD), intact plasma and acrosome membranes (IPAM), intact plasma membrane and damaged acrosome (IPMDA), damaged plasma membrane and intact acrosome (DPMIA), damaged plasma and acrosome membranes (DPAM), high mitochondrial potential (HMP), low mitochondrial potential (LMP), without membrane alteration and stressed (PI–CR+), without membrane alteration and not stressed (PI–CR–), with membrane alteration and stressed (PI+CR+), with membrane alteration and not stressed (PI+CR–), low chromatin resistance sperm (OA+), and high chromatin resistance sperm (OA–) as described by Campanholi et al. (9). In the present study, we added three predictor variables: season, donor, and percentage of viable oocytes. These specific variables were chosen in our study as important attributes of sperm structure, strongly related to fertility, especially in IVEP, as already recognized by the scientific community. The intermediate layers ranged from 1–3 and are composed of neurons; in this study, each layer had 20–300 neurons. The output layer comprised the categories high embryo yield and low embryo yield.

The learning algorithm used for ANN training was backpropagation (37), which is a supervised learning algorithm (38, 39). In this study, the creation of ANNs was performed randomly by varying the number of neurons per layer, transfer functions, and ANN training functions (40). For ANN training and learning, the database was split as follows: 70% for the training set, 15% for the validation set, and 15% for the test set (41–43). Finally, to determine the optimal ANN, we used the genetic algorithm (GA) technique (44). GA is an AI technique that comprises a set of computational algorithms, based on the theory of Darwinian evolution, where genetic operators are used, such as crossing over, mutation and migration, so that sufficient variations occur in the ANN population, in such a way that, after several generations, an optimal ANN is determined (45). The development of the AI software was completely performed on the MATLAB ^®^ platform, which has a specific toolbox for this development (40).

## 3. Results

As a result of the application of the learning algorithm with selection by the GA, three ANN architectures with high performance were obtained, as shown in Table 1. Considering only the training stage, the models correctly classified an average of 81.9% of the data (*n* = 162).

**Table 1:** Predictive capability of artificial neural network (ANN) architectures for embryo yield.

**ANN architecture**	**Correct rating (%)**		
	**High embryo yield**	**Low embryo yield**	**Total**
ANN 1	90.7	90.8	90.7
ANN 2	80.8	70.2	75.3
ANN 3	80.2	78.9	79.6

**Figure 1:**
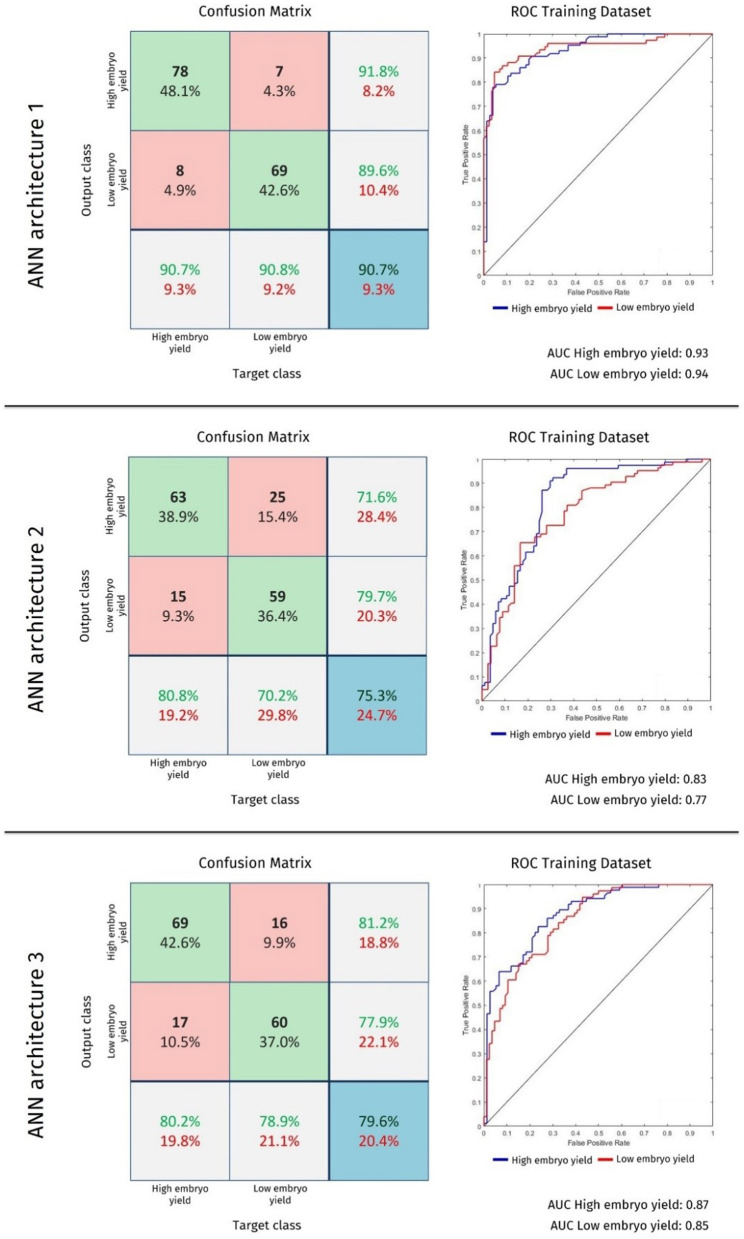
Confusion matrix, receiver operating characteristic (ROC), and area under the curve for ANN 1, 2, and 3 architectures. The y-axis in ROC represents the sensitivity, and the x-axis refers to 1-specificity. In the confusion matrix, the green frames present the number of data and percentage of correctly performed classifications. Gray frames show the ratio of correct and incorrect classifications in each row and column of the array. The blue frame shows the overall error and hit percentage of the ANN model for the blind test data.

Among the three architectures, ANN 1 achieved the best results and correctly predicted the production of 147 embryos. This model also exhibited the highest validation and testing errors (34.3 and 28.6%, respectively). In contrast, ANN 3 did not have the highest predictive ability and the validation and test error rates were the lowest among the three models (25.7 and 22.9%, respectively). The efficiency of the models can be compared using the confusion matrix, ROC analysis, and AUC as shown in Figure 1.

**Figure 2:**
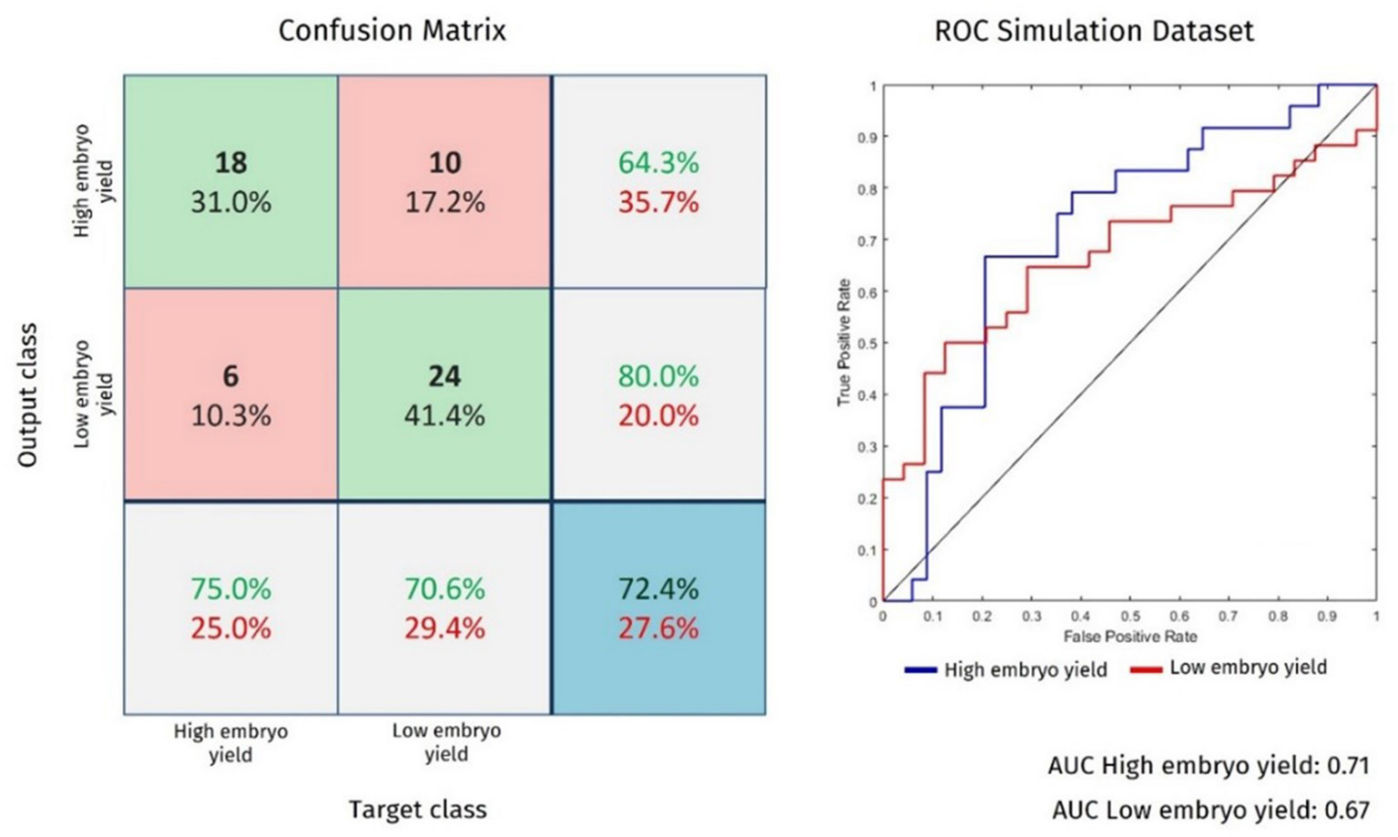
Confusion matrix and ROC curve for architecture 3 when applying blind test data.

Finally, in the blind test (data never analyzed by AI), among all the ANNs, the architecture 3 model accurately predicted 72.4% of the embryo yield (*n* = 42) out of 58 data. The results are shown in Figure 2.

## 4. Discussion

The more sperm characteristics evaluated in a seminal sample, the greater the accuracy obtained about fertility (2). Prediction of fertility in bulls requires knowledge of statistical procedures and reproductive biology to avoid meaningless or erroneous conclusions (46). An instrument that can be used to classify, recognize patterns and make predictions is the use of ANNs. According to Hamadani et al. (47) ANN can offer improving various aspects of animal science by drawing hitherto unknown inferences which were not possible using conventional data analysis techniques. This study is the first in implementing AI through ANN analysis to estimate IVEP in bulls. Different models could be generated to estimate IVEP in Senepol bulls. The dependence relationship between the variables and IVEP is not linear in Senepol bulls; therefore, ANN models fit better in these cases.

Some studies in humans have reported the possibility of estimating fertility using ANN analysis. Neiderberger et al. (48) reported that this methodology can be successfully used to predict fertility potential in humans, since this data analysis showed better results than linear and quadratic discriminant analyses for fertility prediction, which are evaluated based on the sperm penetration assay in bovine cervical mucus and sperm penetration assay in zona-free oocytes. Milewski et al. (49) reported that ANNs predicted the failure of reproductive treatments in patients with almost 90% accuracy. Durairaj and Thamilselvan (50) observed that ANNs predicted the success rate of IVF treatment with 73% accuracy. Milewski et al. (51) created a model using this methodology that correctly predicted approximately 70% of the pregnancies. Siristatidis et al. (19) proposed a functional model for predicting IVF in humans using ANNs to help clinicians modify the treatment plan for subfertile couples and improve the outcome of assisted reproduction.

Similar metrics were observed in our study. In the training stage, the models were able to correctly classify an average of 81.9% of the data, and in the blind test, the model using architecture 3 was able to correctly predict 72.4% of the embryo yield, that is, when it was provided new data (never analyzed before) to the software to predict the output. According to Zaninovic and Rosenwaks (52), the use of AI yields more objective, faster, and accurate results. Similarly, Deb et al. (53) revealed that the prediction efficiency of the ANN model is higher than that of the multiple regression analysis model in predicting the post-thaw motility sperm of bulls.

In animals, ANNs have been used to predict milk production in cows (22, 24) and goats (21), growth in sheep (20), egg production in laying hens (23), birth weight of piglets, number of mummified piglets (25), and body weight of goats (26). However, few studies in animal andrology have used ANNs. One study used this analysis to identify three sperm subpopulations in cat semen based on sperm kinetics and observed the differences in the characteristics of the subpopulations before (sperm from the tail of the epididymis) and after ejaculation [sperm from the ejaculate; (54)]. Deb et al. (53) reported that ANN methodology can be used to predict post-thawing sperm motility in crossbred bulls. Other study recently investigated the determination of the gender of calves using some artificial intelligence techniques, and ANN had 96% of accuracy (55). The increasing use of ANNs in biological studies has been enabled by significant improvements in software, hardware, and methodology, all of which continue to be developed (56). The use of AI permits standardization, automation, and precision in IVEP, generating a lot of enthusiasm for his methodology and even gaining traction in commercial application in human reproductive medicine (52). We believe that our study contributes to expand the applicability of ANNs in research aimed at predicting fertility in different animal species. Analyzing a semen sample before performing IVEP with the ability to predict the outcome of the procedure would tremendously contribute to the selection of better sires and help avoid the high costs of performing biotechnology-based procedures with low embryo yield.

The potential ethical concernings derived from our work are related, primarily, from the needs of improvement of the evaluation process. As a first attempt to approach the problem (in fact, a concept proof) we realized that the software is not excluding the human being on the analysis. Our intending is to produce an objective and reproducible tool to support the human decision on that matter. By now, we are not envisioning the obsolescence of the personal evaluation in favor of the AI system. Currently, all the AI products appearing in animal reproduction research is an attempt to support the human decision with an extra tool (with unbiased, objective, fatigue or bad mood proof and reproducible whereas the laboratory or the team are) but, at least by our knowledge, far from to be a replacement trend.

Although our results were encouraging, we need to emphasize that different types of reproductive strategies may possibly produce different outcomes. Some studies have reported positive correlations between IVEP and *in vivo* fertility in cattle (57–60). However, other studies have observed that the results obtained *in vitro* do not guarantee the same results in the field (61–63). Possibly, the effect of sperm attributes could be different even when the same evaluation is performed to estimate fertility *in vivo* in cattle. In addition, these models need to be validated with a larger number of samples and bulls from other breeds and populations to confirm if these seminal variables can predict embryo yield in a different data set.

In the present study, IVEP was estimated in the Senepol breed using ANNs by combining the evaluation of sperm subpopulations, plasmatic and acrosomal membrane integrity, mitochondrial potential, oxidative status, chromatin resistance, sperm morphology, season, donor, and percentage of viable oocytes. Further studies should be conducted on the application of ANN analysis to estimate fertility outcomes in other reproductive management practices in cattle, such as artificial insemination and natural mating, as well as in other breeds.

## 5. Conclusion

ANN analysis was able to generate models to estimate IVEP in Senepol bulls using 18 seminal variables, season, donor, and percentage of viable oocytes as predictor variables. The model 3 was chosen due its better performance when the blind test was performed over models 1 and 2.

## Data availability statement

The raw data supporting the conclusions of this article will be made available by the authors, without undue reservation.

## Ethics statement

The animal study was approved by FCAV/UNESP Animal Ethics Committee (Protocol No. 12,807/16). The study was conducted in accordance with the local legislation and institutional requirements.

## Author contributions

SC: Conceptualization, Data curation, Investigation, Methodology, Writing—original draft, Writing—review and editing. SG: Resources, Writing—review and editing. GP: Data curation, Formal analysis, Validation, Writing—review and editing. MNo: Data curation, Formal analysis, Writing—review and editing. JR: Data curation, Formal analysis, Validation, Writing—review and editing. JL: Investigation, Writing—review and editing. AS: Investigation, Writing—review and editing. MNi: Investigation, Writing—review and editing. MA: Investigation, Writing—review and editing. AB: Data curation, Resources, Writing—review and editing. FM: Conceptualization, Methodology, Writing—review and editing. LG: Conceptualization, Data curation, Funding acquisition, Project administration, Resources, Supervision, Writing—review and editing.
